# Branched-chain amino acids supplementation induces insulin resistance and pro-inflammatory macrophage polarization via INFGR1/JAK1/STAT1 signal pathway

**DOI:** 10.1186/s10020-024-00894-9

**Published:** 2024-09-12

**Authors:** Huaying Huang, Heye Chen, Yu Yao, Xueyong Lou

**Affiliations:** 1 Department of Endocrinology and Metabolism, JinHua Municipal Central Hospital, No. 365, Renmin East Road, Wucheng District, Jinhua, Zhejiang China; 2 Department of Neurology, JinHua Municipal Central Hospital, Jinhua, 321000 Zhejiang China

**Keywords:** Branched-amino acids, Adipose tissue inflammation, Obesity, Insulin resistance, Pro-inflammatory macrophage polarization, INFGR1/JAK1/STAT1 pathway

## Abstract

**Background:**

Obesity is a global epidemic, and the low-grade chronic inflammation of adipose tissue in obese individuals can lead to insulin resistance and type 2 diabetes. Adipose tissue macrophages (ATMs) are the main source of pro-inflammatory cytokines in adipose tissue, making them an important target for therapy. While branched-chain amino acids (BCAA) have been strongly linked to obesity and type 2 diabetes in humans, the relationship between BCAA catabolism and adipose tissue inflammation is unclear. This study aims to investigate whether disrupted BCAA catabolism influences the function of adipose tissue macrophages and the secretion of pro-inflammatory cytokines in adipose tissue, and to determine the underlying mechanism. This research will help us better understand the role of BCAA catabolism in adipose tissue inflammation, obesity, and type 2 diabetes.

**Methods:**

In vivo, we examined whether the BCAA catabolism in ATMs was altered in high-fat diet-induced obesity mice, and if BCAA supplementation would influence obesity, glucose tolerance, insulin sensitivity, adipose tissue inflammation and ATMs polarization in mice. In vitro, we isolated ATMs from standard chow and high BCAA-fed group mice, using RNA-sequencing to investigate the potential molecular pathway regulated by BCAA accumulation. Finally, we performed targeted gene silence experiment and used immunoblotting assays to verify our findings.

**Results:**

We found that BCAA catabolic enzymes in ATMs were influenced by high-fat diet induced obesity mice, which caused the accumulation of both BCAA and its downstream BCKA. BCAA supplementation will cause obesity and insulin resistance compared to standard chow (STC) group. And high BCAA diet will induce pro-inflammatory cytokines including Interlukin-1beta (IL-1β), Tumor Necrosis Factor alpha (TNF-α) and monocyte chemoattractant protein-1 (MCP-1) secretion in adipose tissue as well as promoting ATMs M1 polarization (pro-inflammatory phenotype). Transcriptomic analysis revealed that a high BCAA diet would activate IFNGR1/JAK1/STAT1 pathway, and IFNGR1 specific silence can abolish the effect of BCAA supplementation-induced inflammation and ATMs M1 polarization.

**Conclusions:**

The obesity mice model reveals the catabolism of BCAA was disrupted which will cause the accumulation of BCAA, and high-level BCAA will promote ATMs M1 polarization and increase the pro-inflammatory cytokines in adipose tissue which will cause the insulin resistance in further. Therefore, reducing the circulating level of BCAA can be a therapeutic strategy in obesity and insulin resistance patients.

**Supplementary Information:**

The online version contains supplementary material available at 10.1186/s10020-024-00894-9.

## Background

Adipose tissue inflammation plays a crucial role in the development of obesity and type 2 diabetes, leading to insulin resistance and metabolic dysregulation (Zatterale et al. 2020). Previously, adipose tissue is regarded as a storage site for energy, it is also now recognized as an active endocrine organ that releases various bioactive substances, including adipokines, cytokines, and chemokines (Coelho et al. 2013). In the context of obesity, the expansion of adipose tissue leads to cellular stress and an unhealthy immune response, resulting in chronic low-grade inflammation (Kawai et al. 2021). This inflammation disrupts the metabolic balance within the adipose tissue and contributes to the insulin resistance seen in type 2 diabetes (Burhans et al. 2018).

The infiltration of immune cells, particularly M1 (pro-inflammatory phenotype) macrophages, into adipose tissue and the release of inflammatory mediators worsen insulin resistance (Surmi and Hasty 2008). These processes reflect a complex interaction between metabolic excess and immune regulation, where over-nutrition and altered adipocyte metabolism in obesity trigger a series of immune responses (Surmi and Hasty 2008). The resulting inflammation impairs insulin signaling pathways, leading to reduced glucose uptake and elevated blood glucose levels (Rehman and Akash 2016). Additionally, the chronic inflammation associated with adipose tissue in obesity contributes to the progressive decline in pancreatic beta-cell function, a key characteristic of type 2 diabetes (Kim et al. 2589). Understanding the mechanisms behind adipose tissue inflammation in obesity and its role in the development of type 2 diabetes is essential for developing targeted therapies to address these widespread metabolic disorders.

Branched-chain amino acids (BCAAs)—leucine, isoleucine, and valine—are essential nutrients that play a pivotal role in muscle protein synthesis and energy production (Wolfe 2017; Holeček 2018). However, beyond their fundamental nutritional value, emerging research has highlighted a more complex relationship between BCAA metabolism and the pathophysiology of metabolic disorders such as obesity and type 2 diabetes (Vanweert et al. 2022). In these conditions, alterations in BCAA catabolism have been observed, with significant implications for systemic metabolism and insulin sensitivity (Blair et al. 2021). In the context of obesity and type 2 diabetes, elevated plasma levels of BCAAs have been consistently reported (Ding et al. 2021; Katagiri et al. 2018). This hyper aminoacidemia is thought to reflect a state of impaired BCAA catabolism rather than increased dietary intake alone. The mechanisms leading to this dysregulation are multifaceted, involving both genetic and environmental factors that affect the enzymes and pathways responsible for BCAA breakdown.

The initial step in BCAA catabolism is a reversible transamination that converts BCAAs into their respective branched-chain a-keto acids (BCKAs) and is catalyzed by the enzyme branched-chain aminotransferase (BCAT), the second step is an irreversible step that BCKA will be further decarboxylation via ketoacid dehydrogenase (BCKD) complex, BCKDH kinase (BDK) and protein phosphatase 1 K (PP2CM) negatively and positively controls the BCKD complex by phosphorylating and dephosphorylating BCKDH-A at serine-293 to control the BCKAs catabolism., leading to the production of C3- and C5-acylcarnitine, acetyl-CoA and succinyl-CoA for the TCA cycle (2, 3). The association between BCAA metabolism and macrophage functions has been reported before. For example, BCKA secreted from tumor cells can modulate phagocytosis of macrophages (Silva et al. 2017) and BCAT1 inhibition reduces oxygen consumption and glycolysis in human macrophages (Papathanassiu et al. 2017). But, whether BCAA catabolism is involved in macrophage polarization and inflammation has never been explored.

Macrophage polarization is subject to a complex interplay of influences, such as cytokines, growth factors, microbial elements, and metabolic cues (Thapa and Lee 2019; Pérez and Rius-Pérez 2022). Pro-inflammatory M1 macrophages arise in response to signals like interferon-γ (IFN-γ) and lipopolysaccharide (LPS), which activate transcription factors including signal transducer and activator of transcription 1 (STAT1) (Chen et al. 2023). This signaling axis is crucial for steering macrophages toward the M1 pro-inflammatory state (Han et al. 2023). The process begins when IFN-γ binds to its receptor, IFNGR1, on the macrophage surface, setting off a series of intracellular events crucial for macrophage activation and immune defense against pathogens and tumors (Nguyen et al. 2001). The binding of IFN-γ to IFNGR1 causes the receptor to change shape, enabling the activation of Janus kinase 1 (JAK1), which is tethered to the receptor's inner domain. JAK1 phosphorylates the receptor, creating attachment points for STAT1 (Sengupta et al. 2019). Once STAT1 is bound and phosphorylated by JAK1, it forms dimers that move into the nucleus. There, the phosphorylated STAT1 dimers bind to γ-activated sequence (GAS) elements in gene promoters, initiating the transcription of genes that drive the M1 macrophage pro-inflammatory responses (Tessitore et al. 1998).

Therefore, in this study, we want to investigate whether disrupted BCAA catabolism will regulate the macrophage polarization and influence the adipose tissue inflammation. The underlying mechanisms will provide us a new insight between amino acids metabolism and macrophage dysfunction and provide us a new therapeutic target to treat obesity and type 2 diabetes in the future.

## Methods

### Animal

Total 40 C57BL/6 J male mice (22 ± 2 g) were purchased from Hunan Slack Jingda Experimental Animal Co., Ltd, they were randomly divided into 3 groups. STC group (n = 20), HFD (n = 10), High BCAA group (n = 10). STC group mice were fed with standard chow for 16 weeks. HFD group mice were fed with a high-fat diet for 16 weeks. High BCAA group was fed with a high BCAA diet (L-Amino Acid Rodent Diet Based on Teklad 7002 Rodent Chow With 150% Added BCAA, Research Diet Inc, catalog #A12030801). The housing conditions for the animals were maintained under specific pathogen-free circumstances, with a controlled temperature of 22 ± 2 °C and a relative humidity of 55% (ranging from 45 to 70%), and under a 12-h light–dark cycle. Mice were sacrificed by CO2 asphyxiation after 16 weeks of experimental period, all procedures involving the animals were performed following China's national regulations on the use of experimental animals and were in line with the standards set by the Laboratory Animal Welfare and Ethics Committee of Jinhua Municipal Central Hospital, which reviewed and approved the guidelines (AL-JHYY202415).

### Histology and immunohistochemical Study

The collected subcutaneous white adipose tissue (sWAT) was fixed by inflation with a 4% buffered paraformaldehyde solution (dissolve in water, v/v). The tissues were then embedded in paraffin, after dehydration, cut into sections (5 μm) and stained using Hematoxylin & Eosin (H&E), anti-TNF-α, anti-MCP-1 and anti-IL-1β antibody. These stained sections were examined under a light microscope and analyzed using the Image J software. The antibodies used for TNF-α staining were sourced from Abcam (Catalog #ab220210) at a dilution of 1/200, for IL-1β staining from Abcam (Catalog #ab205924) at a dilution of 1/200, and for MCP-1 staining from Abcam (Catalog #ab214819) at a dilution of 1/200.

### Measurement of cytokines

Adipose tissue samples were collected and rapidly frozen in liquid nitrogen. Upon experimentation, the tissues were homogenized in 500 µL of 0.2 M sodium acetate solution (pH 4.5) using a Precellys tissue homogenizer. The homogenate was centrifuged at 800*g* for 10 min at room temperature to remove debris, and the resulting supernatant was used to measure TNF-α (Catalog# DY410, R&D Systems), IL-1β (Catalog# DY401, R&D Systems), and MCP-1 (Catalog# DY479, R&D Systems) by using commercial ELISA kits.

### Immunofluorescence staining

For immunofluorescence staining, antigen retrieval was performed on the sections by boiling them in sodium citrate buffer (0.1 mol/L sodium citrate, 0.1% Tween 20, pH 6.0) for 20 min. This was followed by blocking with 5% FBS in PBS for 1 h at room temperature. The sections were then incubated overnight at 4 °C with mouse anti-F4/80 (Abcam, Cat#ab6640, 1/500 dilution) and anti-iNOS (Abcam, Cat#ab178945, 1/500 dilution) antibodies. The next day, fluorescent dye-conjugated secondary antibodies (anti-rabbit 594 nm and anti-mouse 488 nm) were applied at room temperature for 1 h. Finally, the sections were mounted using ProLong™ Glass Antifade Mountant (Catalog# P36980, Invitrogen). The distribution of fluorescence in sWAT was observed using a laser scanning confocal microscope (Leica TCS SP8 CARS, Germany).

### Glucose tolerance test and insulin tolerance test

Mice were fasted for 12 h under glucose tolerance test in 15 weeks. After measuring the baseline blood glucose concentration from a tail cut by a Glucometer test strip, mice were injected intraperitoneally with 20% glucose at 1.5 mg/g body weight. Blood glucose concentrations were measured at 15, 30, 45, 60, 90, and 120 min after glucose injection. Mice were fasted for 6 h under insulin tolerance test in 16 weeks. After measuring the baseline blood glucose concentration, mice were injected intraperitoneally with recombinant human insulin at 1.2 mU/g body weight (Catalog# 91,077 C, Sigma–Aldrich). Blood glucose concentrations were then measured at 15, 30, 45, 60, 90, and 120 min after insulin administration.

### Isolation of adipose tissue macrophages

To procure visceral adipose tissue, we meticulously excised any apparent blood vessels and connective tissue using sterilized forceps and scissors. We then measured out two grams of this tissue, rinsed it thoroughly with 20 mL of Dulbecco's Phosphate-Buffered Saline (DPBS), and finely chopped it into pieces ranging from 1 to 2 mm in size. Following this, we subjected the chopped tissue to centrifugation at 550*g* in DPBS at a temperature of 4 °C for 10 min, which served to eliminate any red blood cells. Afterward, we introduced a digestion solution to the tissue and maintained the mixture at 37 °C for an hour to facilitate digestion. We then filtered the digested material through a 70 µm strainer (Catalog #CLS431751, Merck), performed another round of centrifugation, and collected the resultant pellet. This pellet was further processed to extract macrophages, utilizing CD14 + magnetic beads (Invitrogen, Catalog# 11149D) in alignment with the guidelines provided by the bead manufacturer. Once isolated, we quantified the macrophages, evaluated their viability, and prepared them for plating, setting the stage for further experimental investigations.

### Flow cytometry

Isolated adipose tissue macrophage was analyzed using FACS assays on an LSRFortessa instrument (BD Biosciences). Subsequent statistical analysis of the data was performed using FlowJo software. For staining, cells were incubated with fluorochrome-conjugated antibodies, specifically anti-F4/80 (Abcam, Catalog #ab6640, at a dilution of 1/500), anti-206 (Abcam, Catalog #ab64693, at a dilution of 1/500), anti-CD86 (Abcam, Catalog #ab239765, at a dilution of 1/500), anti-11b (Abcam, Catalog #ab133357, at a dilution of 1/500).

### RNA isolation

Total RNA was isolated using TRIzol reagent (Catalog# 15,596,026, Invitrogen), and reverse transcription of 1 μg of this RNA was conducted utilizing the GoScript™ Reverse Transcription Mix (Catalog# A2801, Promega). Subsequent real-time quantitative PCR (QPCR) analyses were carried out on an Applied Biosystems ViiA 7 Real-Time PCR System, employing QuantiNova SYBR® Green PCR Kit (Catalog# 208,056, QIAGEN) alongside specific primers for various genes as listed in Supplementary Table 1.

### RNA‑sequencing

Total RNA was isolated using the RNeasy mini kit (Qiagen). Strand-specific RNA-seq libraries were prepared with the TruSeq stranded total RNA sample preparation kit (Illumina), quantified by the Qubit 2.0 Fluorometer (Life Technologies), and assessed for insert size with the 2100 bioanalyzer (Agilent). Clusters were generated on the cBot at a concentration of 10 pM and sequenced on the NovaSeq 6000 system (Illumina). Library preparation and sequencing were carried out by the Meiji Biotechnology Corporation.

#### KEGG enrichment analysis and gene set enrichment analysis (GSEA)

Differentially expressed genes (DEGs) were identified using DESeq2 from RNA-seq data. The gene list was filtered based on an adjusted P-value < 0.05 and a log2 fold change > 1. The resulting gene symbols were converted to Entrez Gene IDs using the org.Hs.eg.db package in R. KEGG Pathway Enrichment: KEGG pathway enrichment analysis was performed using the clusterProfiler package in R. The Entrez Gene IDs were input into the enrichKEGG function, specifying the organism as Homo sapiens (hsa). The significance of pathway enrichment was determined using a hypergeometric test, and P-values were adjusted for multiple testing using the Benjamini–Hochberg method. Pathways with an adjusted P-value < 0.05 were considered significantly enriched. Visualization of enriched pathways was conducted using the dotplot function.

GSEA was performed using the fgsea package in R. The ranked gene list and gene sets were input into the fgsea function. The analysis was run with a minimum gene set size of 15 and a maximum size of 500. Enrichment scores were calculated, and P-values were adjusted using the Benjamini–Hochberg method. Gene sets with an adjusted P-value < 0.05 were considered significantly enriched. Enrichment plots were generated for visualization.

#### Protein analysis by immunoblotting

Cellular or tissue samples were lysed in RIPA buffer (comprising 150 mM NaCl, 50 mM Tris–HCl at pH 7.4, 2 mM EDTA, 0.1% SDS, and 1% NP-40), which was fortified with a protease inhibitor mix (Catalog# HY-K0010, MedChemExpress) and a phosphatase inhibitor cocktail (Catalog# B15001, Bimake). The lysates were then subjected to protein separation by SDS-PAGE and the proteins were subsequently transferred to a PVDF membrane (Catalog# 1,620,177, BIO-RAD) with 300 mA for 2 h. Blocking of the membrane was performed using 10% nonfat milk for one hour, after which it was incubated with the appropriate primary antibody at 4 °C overnight. Following primary antibody incubation, the membrane was washed with TBST (containing 2.7 mM Tris base, 137 mM NaCl, and 0.1% Tween 20) and incubated with the relevant HRP-conjugated secondary antibody from Cell Signaling Technology for one hour at ambient temperature. Post-secondary antibody incubation, the membrane was washed again with TBST, and the protein bands were detected using ECL reagents (BIO-RAD), with band intensity quantification performed via ImageJ software. Anti-PP2CM antibody (catalog #ab135286, abcam), Anti-BCAT1 antibody (catalog # ab232700, abcam), Anti-BCAT2 antibody (catalog # ab307833, abcam), Anti-BCKDH-A antibody (catalog # ab305168, abcam), Anti-BDK antibody (catalog # ab128935, abcam), Anti-phospho-BCKDH-A antibody (catalog # ab302504, abcam), Anti-beta Actin antibody (catalog #ab8226, abcam), Anti-iNOS antibody (catalog # ab283655, abcam), Anti-JAK1 antibody (catalog # ab133666, abcam), Anti-phospho-JAK1 antibody (catalog # ab138005, abcam), Anti-SIRT1 antibody (catalog # ab189494, abcam), Anti-phospho-SIRT1 antibody (catalog # ab76029, abcam), Anti-IFGR1 antibody (catalog #12–5945-82, Invitrogen), Anti-IFGR2 antibody (catalog #PA5-109,847, Invitrogen).

#### Small interfering RNA (siRNA) transfection

siRNAs (GenePharma) were mixed with DharmaFECT 3 transfection reagent (Catalog# T-2003–03, Dharmacon) followed the manufacturer’s instruction and added to the macrophages at a final concentration of 20 nM for 48 h. The sequence of si-IFNGR1 is 5’-CCGGGCCAGAGTTAAAGCTAAGGTTCTCGAGAACCTTAGCTTTAAC.

TCTGGCTTTTTG-3’ (SHCLNG-NM_010511), IFNGR1 siRNA-Sigma.

#### Statistical analysis

Data were processed using GraphPad Prism 8.0.1 software. The Kaplan–Meier survival analysis with a log-rank test was used for the horizontal grid test comparisons. Histological data were analyzed using the Mann–Whitney test. Two-way ANOVA with Tukey’s multiple comparison test was applied for other comparisons. P-value < 0.05 was considered as statistical significance.

## Results

### BCAA catabolism in adipose tissue macrophages is altered in HFD-induced obesity mice.

**Fig. 1:**
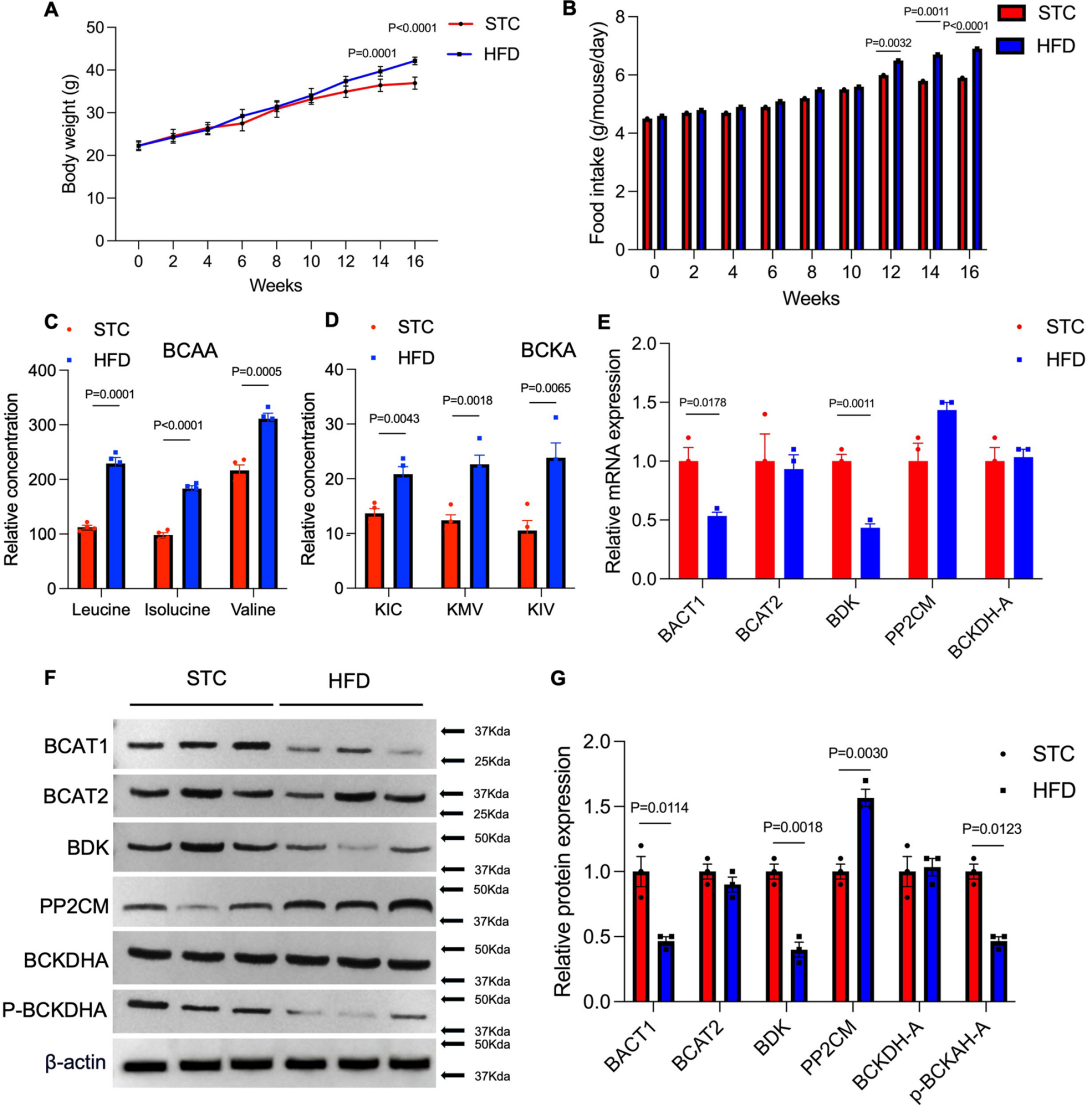
BCAA catabolism in adipose tissue macrophages is altered in HFD-induced obesity mice. 20 C57B/L mice (8 weeks around 22 g) male mice were randomly divided into 2 groups. STC group was fed with standard chow diet, HFD group were fed with high-fat diet (60 kcal% Fat), the experiment were continual 16 weeks. **A**, **B**. body weight and food intake of each group was recorded every 2 weeks. **C** After sacrificed, subcutaneous white adipose tissue (sWAT) were isolated, and adipose tissue macrophage were used to detect the cellular concentration of BCAA (valine, isoleucine and leucin) and (**D**) BCKA including KIC (ketoisocaproate), KMV (keto-β-methyl valerate) and KIV (ketoisovalerate) by LC/MS/MS. **E** the total RNA of adipose tissue macrophage were extracted and were used to detected the mRNA expression of BCAA catabolic enzymes, normalized by the expression of GADPH. **F** total proteins were used to detect the protein expression of BCAA catabolic enzymes. **G** quantitively analyses were showed in the figure. Abbreviation: Branched chain amino acid transaminase 1/2 (BCAT1/2), protein phosphatase 1 K (PPM1K), branched-chain α-ketoacid dehydrogenase-A (BCKDH-A), BCKDH kinase (BDK)

20 C57B/L mice (8 weeks around 22 g) male mice were randomly divided into 2 groups. The STC group was fed a standard chow diet, the HFD group was fed a high-fat diet, and the experiment continued for 16 weeks. Every 2 weeks, the body weight and food intake were recorded. After 16 weeks, we sacrificed the mice from the 2 groups and isolated the adipose tissue macrophage for analysis. The body weight of the HFD group was significantly higher (14 weeks P = 0.0001 and 16 weeks P < 0.0001) than the STC group (Fig. 1A), which suggested the HFD-induced obesity mice model has been established successfully. There was higher food intake in the HFD group than the other group too (Fig. 1B). Then the adipose tissue macrophage was isolated and the cellular BCAA and BCKA were extracted for LC/MS/MS measurement, the results showed that in HFD-induced obesity mice, there were the accumulation of both BCAA (Leucine, Isoleucine and Valine) and BCKA (KIC, KMV and KIV) in adipose tissue macrophage (Fig. 1C, D). We have detected the mRNA expression of BCAA catabolic enzymes, the QPCR results indicated that the mRNA levels of BCAT1, BDK, and P-BCKDHA were decreased significantly, and the mRNA expression of PP2CM was significantly increased (Fig. 1E). The results from immunoblotting experiment were consistent with QPCR results (Figs. 1F), the expression of BCAT1, BCAT2,BDK and phosphorylated BCKDH-A were decreased after BCAA supplementation and the expression of PP2CM was increased (Fig. 1G). These results showed that BCAA catabolism in adipose tissue macrophages was disrupted in HFD-induced obesity mice.

### BCAA supplementation induces obesity and insulin resistance in mice.

**Fig. 2:**
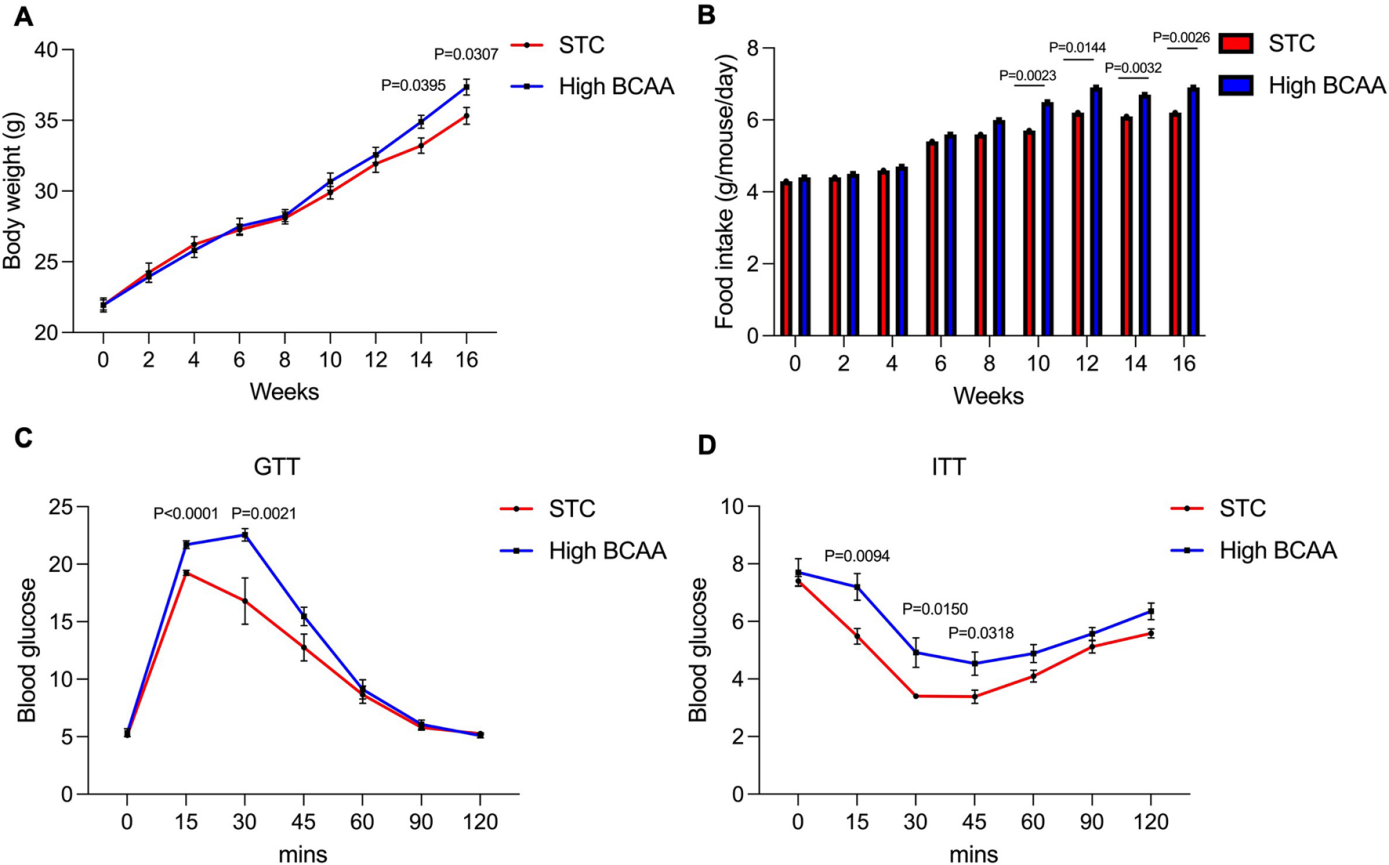
BCAA supplementation induce obesity and insulin resistance in mice. 20 C57B/L mice (8 weeks around 22 g) male mice were randomly divided into 2 groups. STC group was fed with normal diet, High BCAA group was fed with BCAA supplementation diet (With 150% Added BCAA) for 16 weeks. **A**, **B** body weight and food intake of each group were recorded every two weeks. **C** glucose tolerance test was conducted in 15 weeks. mice were fasted for 12 h. After measuring the baseline blood glucose concentration from a tail cut by a Glucometer test strip, mice were injected intraperitoneally with 20% glucose at 1.5 mg/g body weight. Blood glucose concentrations were then measured at 15, 30, 45, 60, 90 and 120 min after glucose injection. **D** insulin tolerance test was conducted in 16 weeks. mice were fasted for 6 h. After measuring the baseline blood glucose concentration, mice were injected intraperitoneally with recombinant human insulin at 1.2 mU/g body weight. Blood glucose concentrations were then measured at 15, 30, 45, 60, 90 and 120 min after insulin administration

20 C57B/L mice (8 weeks around 22 g) male mice were randomly divided into 2 groups. The STC group was fed with normal diet, High BCAA group was fed with BCAA supplementation diet for 16 weeks. The body weight of each group was recorded every 2 weeks. In 14 weeks (P = 0.0395) and 16 weeks (P = 0.0307), we can see significantly increased body weight in the high BCAA group (Fig. 2A). There was a higher food intake of the High BCAA group than STC group (Fig. 2B). And in 15 and 16 weeks, we performed glucose tolerance experiment and insulin sensitivity experiment respectively, the results showed that high BCAA group has poor glucose tolerance compared to the STC group (Fig. 2C). And high BCAA supplementation caused lower insulin sensitivity compared to the STC group (Fig. 2D), which implied that high BCAA supplementation would cause insulin resistance in mice.

### BCAA supplementation induces adipose tissue inflammation.

**Fig. 3:**
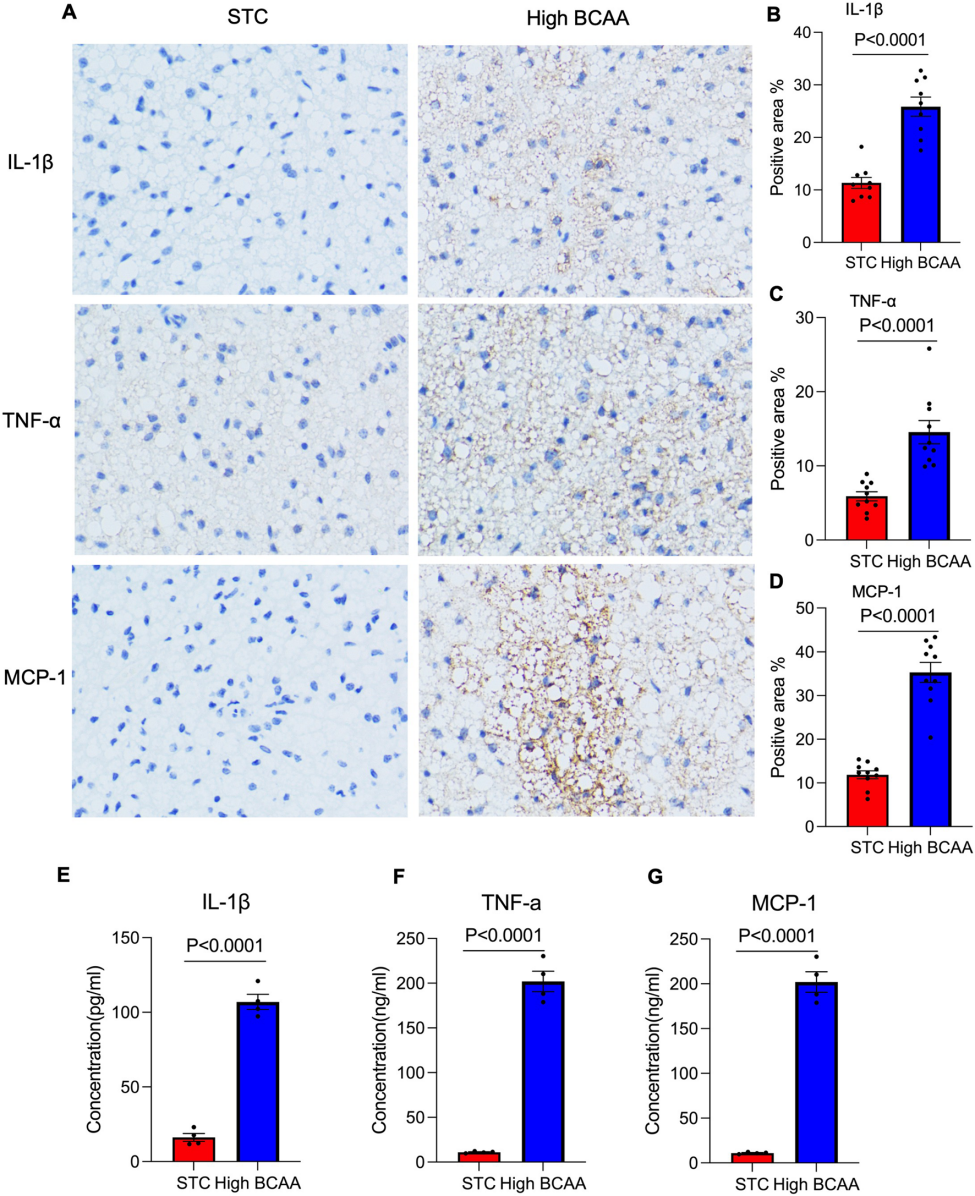
BCAA supplementation induces adipose tissue inflammation. 20 C57B/L mice (8 weeks around 22 g) male mice were randomly divided into 2 groups. STC group was fed with normal diet, High BCAA group was fed with BCAA supplementation diet (With 150% Added BCAA) for 16 weeks. After mice were sacrificed in 16 weeks, (**A**) the subcutaneous white adipose tissue (sWAT) was used to do Immunohistochemical (IHC) Study staining by using anti-IL-1β, anti-TNF-α and MCP-1 antibody at a dilution of 1:200, respectively. **B**–**D** the quantitively analysis of each positive area were attached on right, n = 10. **E** The supernatant of adipose tissue were used to detect the concentration of IL-1β. **F** TNF-α and (**G**) MCP-1 by commercial ELISA kit, n = 4. Data are displayed as mean ± SEM. Statistical significance was tested using two-tailed student’s t-test

Following a 16-week study period, the mice were euthanized, and their subcutaneous white adipose tissue (sWAT) was collected for immunohistochemical analysis. The staining results revealed that the group receiving high levels of branched-chain amino acid (BCAA) supplementation exhibited a more pronounced presence of pro-inflammatory markers IL-1β, TNF-α, and MCP-1 compared to the standard treatment control (STC) group, as depicted in Fig. 3A. Quantitative analysis confirmed a significant upregulation of these pro-inflammatory cytokines in the high BCAA group (P < 0.0001), shown in Figs. 3 B-D. Additionally, ELISA assays conducted on the adipose tissue confirmed the elevated expression of IL-1β, TNF-α, and MCP-1 in the high BCAA-supplemented mice (P < 0.0001), as illustrated in Figs. 3E–G. These findings suggest that BCAA supplementation may induce increased inflammation in adipose tissue.

### BCAA supplementation promotes adipose tissue macrophage M1 polarization.

**Fig. 4:**
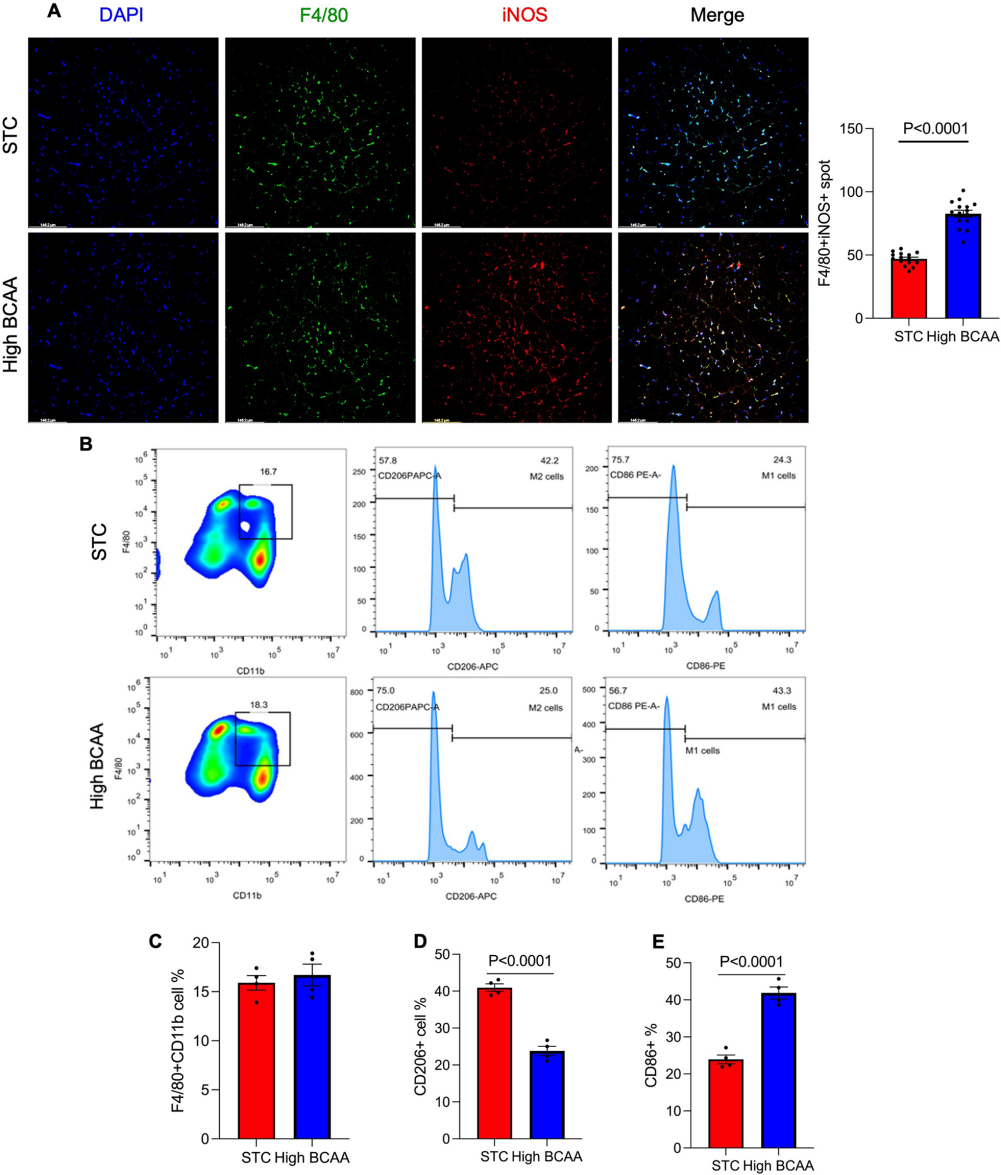
BCAA supplementation promotes adipose tissue macrophage M1 polarization. 20 C57B/L mice (8 weeks around 22 g) male mice were randomly divided into 2 groups. STC group was fed with normal diet, High BCAA group was fed with BCAA supplementation diet (With 150% Added BCAA) for 16 weeks. After mice were sacrificed in 16 weeks, (**A**) the sWAT was used to perform immunofluorescence staining of of F4/80 (green) and iNOS (red). The nucleus was stained with DAPI (blue). Scale bar: 50 μm. Quantification of number of F4/80 + iNOS + cells are displayed in the right panel. Representative images are shown, n = 15. **B** Isolated adipose tissue macrophages were stained by anti-F4/80, anti-CD11b, anti-CD206 and anti-CD86 antibody, follow by the analysis of flow cytometry, n = 4. The quantitively analysis of the (**C**) F4/80 + CD11b + %, (**D**) CD206 + % and (**E**) CD86 + % were attached below. Data are displayed as mean ± SEM. Statistical significance was tested using two-tailed student’s t-test

Since adipose tissue macrophage is the mainly source of pro-inflammatory cytokine, then we examined the macrophage polarization in different groups. sWAT was used to do immunofluorescence staining, we co-stained the DAPI (nucleus marker), F4/80 (macrophage marker) and iNOS (pro-inflammatory macrophage marker). The IF staining results showed that F4/80 signal was no different in the two groups, but the signal of iNOS was significantly increased in the high BCAA group (Fig. 4A), which indicated that BCAA supplementation can promote adipose tissue macrophage pro-inflammatory polarization. To verify our finding, we also performed a flow cytometry experiment, after adipose tissue macrophage isolation, we co-stained the cell with F4/80, CD11b (macrophage marker), CD206 (anti-inflammatory macrophage marker) and CD86 (pro-inflammatory macrophage marker) (Fig. 4B). The results were consistent with IF staining, the population of total macrophage (F480 + CD11b +) showed no change between the two groups (Fig. 4C), but CD206 + macrophage decreased (from 42.2 to 25.0%) and CD86 + macrophage increased (from 24.3 to 43.3%) in the high BCAA group (Fig. 4D, E). These results indicated that BCAA supplementation can promote adipose tissue macrophage from an anti-inflammatory phenotype to a pro-inflammatory phenotype, contributing to the increased levels of pro-inflammatory cytokines observed in adipose tissue after BCAA supplementation.

### RNA-seq analysis of adipose tissue macrophage after BCAA supplementation.

**Fig. 5:**
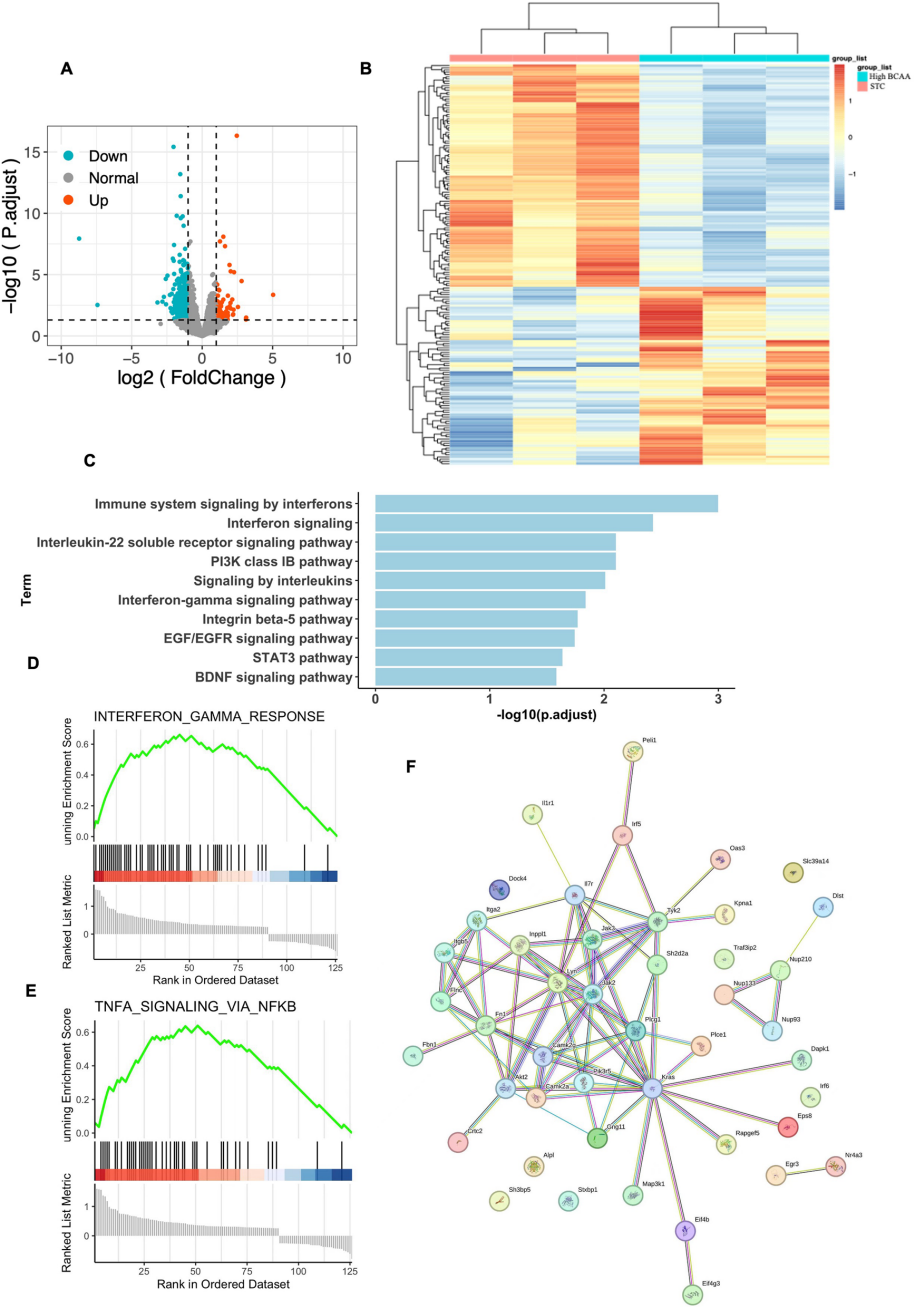
RNA-seq analysis of adipose tissue macrophage after BCAA supplementation. Adipose tissue macrophage isolated from 2 groups were used to perform RNA-seq analysis. **A** the differential expressed genes (DEGs) were identified between two groups, the up regulated genes after BCAA supplementation (right) and down regulated genes after BCAA supplementation (left) were showed. **B** The DEGs between two groups were showed in heatmap. **C** the Kyoto Encyclopedia of Genes and Genomes (KEGG) enrichment analysis of DEGs were enriched on the top 10 signal pathway were showed. **D** Gene Set Enrichment Analysis (GSEA) showing that interferon gamma response and (**E**) TNFa signaling via nfkb were the top enriched pathway after BCAA supplementation in adipose tissue macrophage RNA-seq. **F** The hub genes after BCAA supplementation were identified and we performed the protein–protein interaction (PPI) analysis to reveal the connected map between these hub genes

To investigate the molecular signal pathway activated by BCAA supplementation, adipose tissue macrophages were isolated from mice in both the STC and BCAA supplementation groups. Then RNA-seq was performed to reveal changes in the mRNA profile. The transcriptomic analysis identified 393 differentially expressed genes (DEGs), with 80 genes upregulated and 313 genes downregulated (Fig. 5A, B). Then we used these DEGs to perform KEGG enrichment analysis. The results showed most DEGs enriched on immune system signaling by interferons, Interferon signaling, IL-22 soluble receptor signaling pathway, PI3K class IB pathway, interferon-γ signaling pathway, Integrin beta-5 pathway, EGF/EGFR signaling pathway, STAT3 pathway and BDNF signaling pathway (Fig. 5C). Gene Set Enrichment Analysis (GSEA) indicated that BCAA supplementation activated the IFN-γ and TNF-α pathways (Fig. 5D, E). Additionally, the protein–protein interaction (PPI) network provided a platform to identify disease-related genes based on their functional relationships (Fig. 5F).

### BCAA supplementation activate IFNGR1/JAK1/STAT1 signal pathway.

**Fig. 6:**
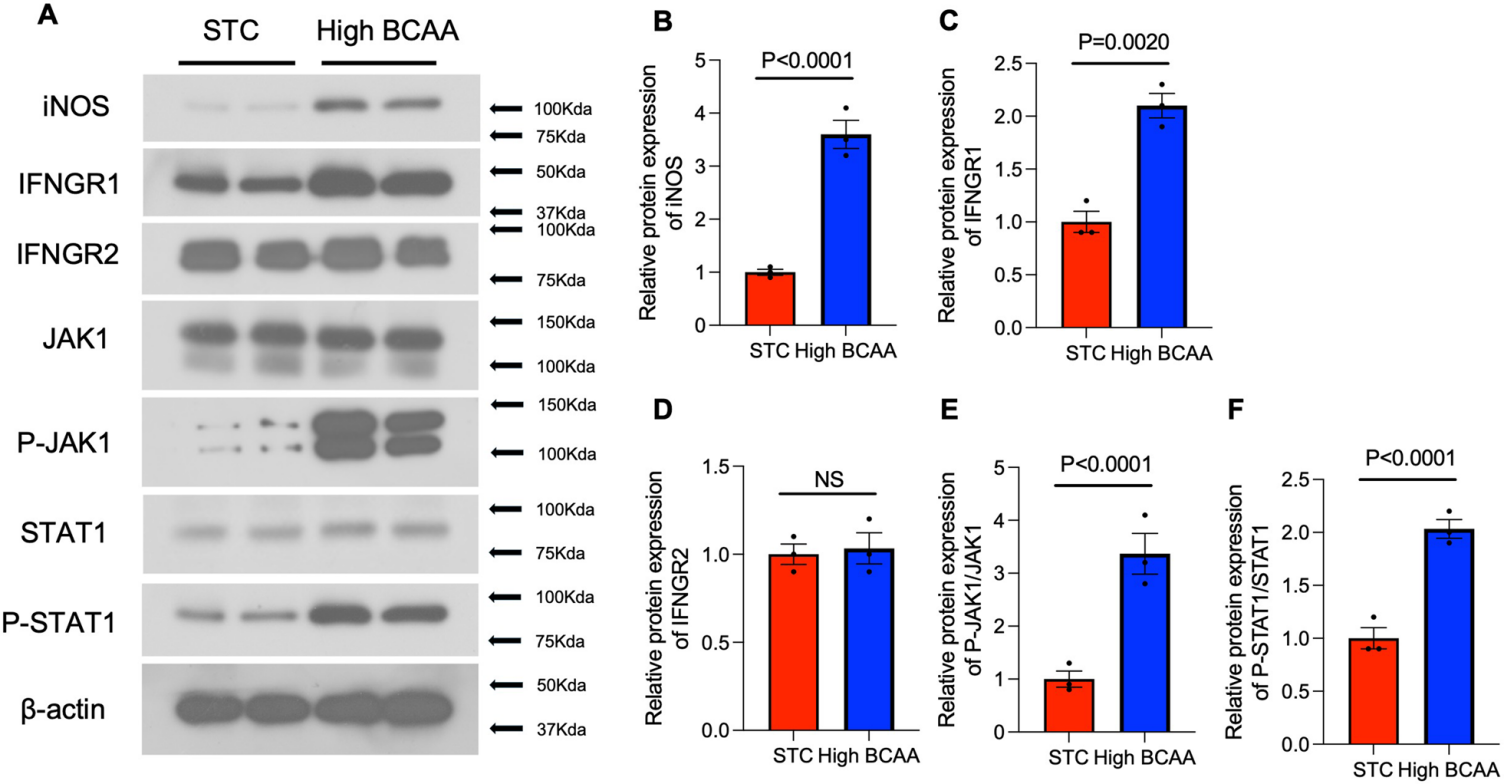
BCAA supplementation induce adipose tissue inflammation and pro-inflammatory macrophage polarization via activating IFNGR1/JAK1/STAT1 signal pathway. Adipose tissue macrophage isolated from 2 groups were used to extract the total protein. **A** the extracted protein samples were used to perform immunoblotting analysis to detect the protein expression of iNOS, IFNGR1, INFGR2, JAK1, P-JAK1, STAT1, P-STAT1 and β-actin, (**B**–**F**) the quantitively analysis of different protein expression were attached on the right, normalized by the expression of β-actin, n = 4. Data are displayed as mean ± SEM. Statistical significance was tested using two-tailed student’s t-test

The RNA-seq data has suggested that BCAA supplementation may contribute to regulating the interferon-γ signaling pathway (Fig. 5D). To verify whether BCAA supplementation can activate this pathway, further experiments were conducted. Adipose tissue macrophages were isolated from the two groups, and the protein expression of iNOS indicated that BCAA supplementation can induce pro-inflammatory macrophage polarization (Fig. 6A, B), consistent with previous results (Fig. 4A). Next, protein expression in the IFN-γ signaling pathway was examined. INF-γ recognizes cell membrane receptors IFNGR1 and/or IFNGR2. Western blot analysis showed that BCAA supplementation increased the protein expression of IFNGR1 but not IFNGR2 (Fig. 6A, C and D). Additionally, the expression of JAK1, P-JAK1, STAT1, and P-STAT1 proteins was assessed. The results demonstrated that BCAA supplementation enhanced the P-JAK1/JAK1 and P-STAT1/STAT1 ratios (Fig. 6E, F). All above results indicated that BCAA supplementation can activate IFNGR1/JAK1/STAT1 signal pathway and regulate adipose tissue macrophage polarization.

### IFNGR1 silence can reverse the effect of BCAA supplementation-induced adipose tissue inflammation and pro-inflammatory macrophage polarization.

**Fig. 7:**
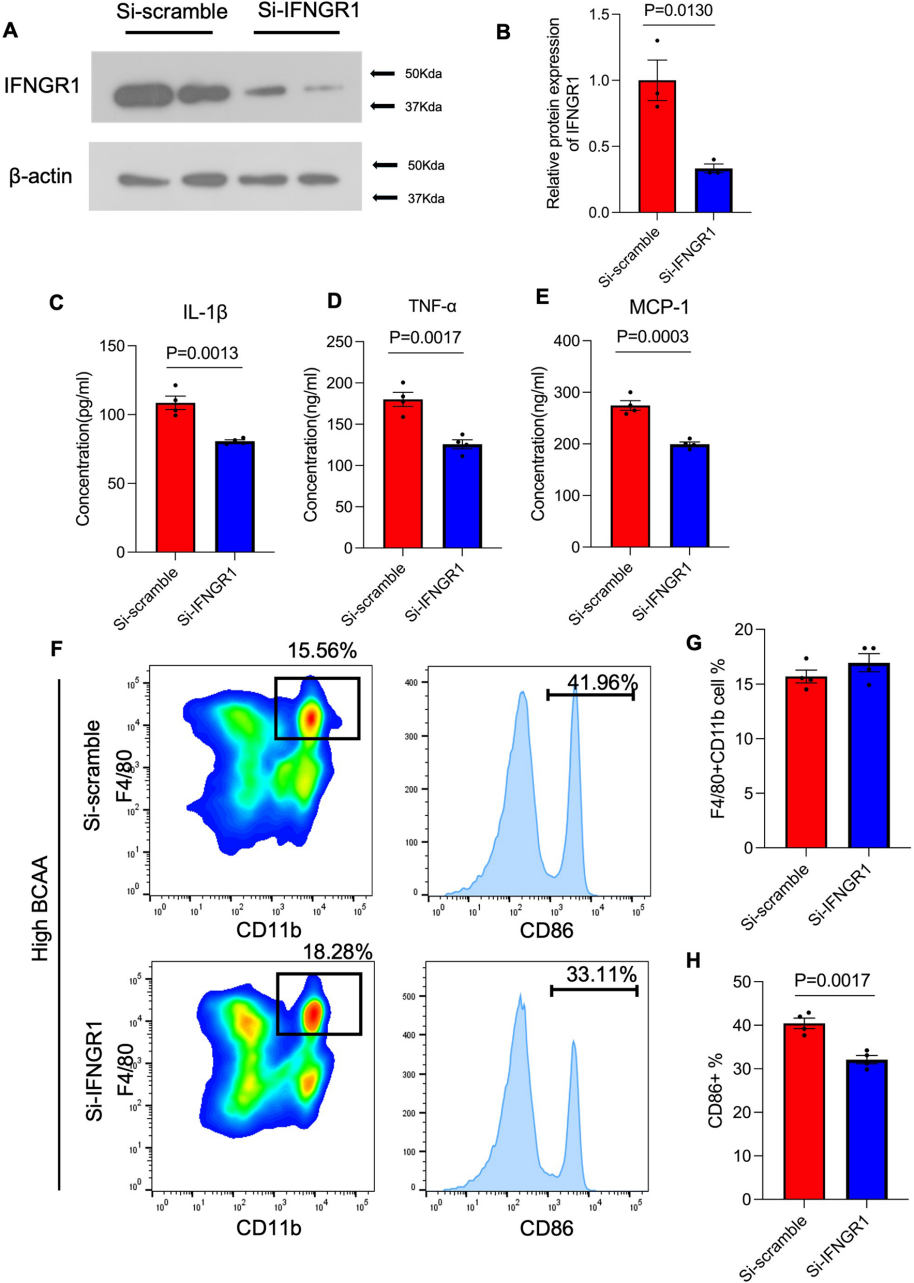
IFNGR1 Silence can reverse the effect of BCAA supplementation-induced adipose tissue inflammation and pro-inflammatory macrophage polarization. Isolated adipose tissue macrophages from BCAA supplementation mice were transfected with si-Scramble as control or si-IFNGR1 for 48 h, then the protein expression of IFNGR1 in two groups were detected by western-blot, the quantitively analysis of the expression of IFNGR1 were attached on the right, normalized by the expression of β-actin, n = 4. **C** after IFNGR1 knockdown, the pro-inflammatory cytokines IL-1β. **D** TNF-α and (**E**) MCP-1 in the supernatant of culture medium were measured by commercial ELISA kit, n = 4 (**F**) adipose tissue macrophages were stained by an-F4/80 (macrophage marker) and anti-CD86 (M1 macrophage marker) antibody, followed by the flow cytometry analysis, the quantitively analysis of (**G**) F4/80 + CD11b positive cell population and (H) CD86 positive population were attached on the right, n = 4

To verify the IFNGR1/JAK1/STAT1 signal pathway is the mainly pathway of BCAA supplantation’s function, adipose tissue macrophages were isolated, and IFNGR1 was silenced using siRNA. Immunoblotting results indicated that the knockdown efficiency of siRNA achieved approximately 60% reduction in IFNGR1 expression (Fig. 7A, B). Subsequently, pro-inflammatory cytokines (IL-1β, TNF-α, and MCP-1) in the culture medium were measured. ELISA results showed that IFNGR1 knockdown inhibited the secretion of these pro-inflammatory cytokines following high BCAA stimulation (Fig. 7C–E). And IFNGR1 knockdown also inhibited the pro-inflammatory macrophage polarization cause by BCAA supplementation (Fig. 7F), there was no change of F4/80 + CD11b + (total macrophage), but significantly inhibited the CD86 + cell (pro-inflammatory macrophage) from 41.96 to 33.11% (Fig. 7G, B). The above results suggested that IFNGR1 Silence can reverse the effect of BCAA supplementation-induced adipose tissue inflammation and pro-inflammatory macrophage polarization. Thus, IFNGR1 serves as a critical target for the high BCAA stimulus.

## Discussion

Chronic low-grade inflammation in adipose tissue is a hallmark of obesity and type 2 diabetes (T2D), and adipose tissue macrophage plays an important role in this process as it is the mainly source of pro-inflammatory cytokines. The role of adipose tissue macrophages in adipose tissue inflammation and obesity is an area of growing research interest with important implications for understanding the pathophysiology of obesity and its associated metabolic complications (Lumeng et al. 2007). In the lean state, adipose tissue is populated by a resident population of macrophages that maintain tissue homeostasis and exhibit an anti-inflammatory phenotype (Suárez-Zamorano et al. 2015). However, with the expansion of adipose tissue during obesity development, the tissue microenvironment undergoes significant changes, leading to a pro-inflammatory milieu (Russo and Lumeng 2018). As obesity progresses, there is a marked increase in the recruitment of circulating monocytes that differentiate into macrophages within the adipose tissue (Li et al. 2023; Boutens and Stienstra 2016). These newly recruited adipose tissue macrophages often display a pro-inflammatory phenotype, characterized by the production of inflammatory cytokines such as tumor necrosis factor-alpha (TNF-α), interleukin-6 (IL-6), and interleukin-1beta (IL-1β) (Hernández-Rodríguez et al. 2004). The shift towards a pro-inflammatory profile is associated with the development of systemic insulin resistance, a key feature of metabolic syndrome and type 2 diabetes.

Our results show that the BCAA catabolism in adipose tissue macrophage is altered in HFD-induce obesity mice and BCAA supplementation can promote adipose tissue macrophages pro-inflammatory polarization and increase the adipose tissue inflammation, we also observe the obesity and insulin resistance in mice after BCAA supplementation. These evidence make it is believed that BCAAs catabolism may contribute to the adipose tissue macrophage function and inflammation, further exacerbating insulin resistance and metabolic dysfunction. The association between high BCAA levels, obesity, and T2DM suggests that BCAAs could potentially serve as early biomarkers for metabolic disease risk. Monitoring BCAA levels could potentially help identify individuals at risk of developing obesity and T2DM, allowing for early intervention. Moreover, understanding the role of BCAAs in these diseases could provide insights into their pathophysiology, offering new avenues for treatment and management.

Besides, we also aim to investigate the underlying mechanism of how elevated BCAA regulate the macrophage polarization. Macrophage polarization is influenced by a complex interplay of factors, including cytokines, growth factors, microbial elements, and metabolic reprogramming (Yao et al. 2019; Cutolo et al. 2022; Liu et al. 2022; Chen et al. 2022). Pro-inflammatory M1 macrophages are induced by IFN-γ and LPS, which activate transcription factors like NF-κB and STAT1 (Lin et al. 2020; Cao et al. 2023). There are numerous studies prove that high BCAA level can activate the NF-κB signal pathway, but our results also provide the evidence that STA1 signal pathway also involved in the inflammatory response of adipose tissue macrophage under high BCAA status. The IFNGR1/JAK1/STAT1 axis begins when IFN-γ binds to its receptor, IFNGR1, on the macrophage surface, triggering a series of intracellular events crucial for macrophage activation and immune defense against pathogens and tumors (Du et al. 2021). The binding of IFN-γ to IFNGR1 causes a conformational change in the receptor, enabling the activation of Janus kinase 1 (JAK1), which is associated with the receptor's intracellular domain. JAK1 phosphorylates the receptor, creating docking sites for STAT1. Once STAT1 is recruited and phosphorylated by JAK1, it forms dimers that translocate into the nucleus, initiating the transcription of genes that drive the M1 macrophage pro-inflammatory responses (Ma et al. 2018; Lu et al. 2023). When we knockdown the expression of IFNGR1, the inflammatory response after BCAA supplementation is reversed, which indicate that IFNGR1 is a critical target for the high BCAA stimulus. As we gain more insight into this pathway, it may present new avenues for therapeutic intervention to modulate macrophage activity in various conditions, including autoimmune diseases, infections, and cancer.

## Conclusions

Our findings provide new insights into the function of BCAA in regulating adipose macrophages and will contribute to clinical dietary intervention and diversified treatment strategies for patients with obesity and type 2 diabetes.

## Supplementary Information

Additional file 1

## Data Availability

The RNA-seq raw data are available upon request to interested researchers.

## Abbreviations

BCAA: Branched-chain amino acids
BCKA: Branched-chain a-keto acids
mTOR: Mammalian target of rapamycin
EDTA: Ethylenediaminetetraacetic acid
H&E: Hematoxylin & Eosin
DPBS: Dulbecco's Phosphate-Buffered Saline
PVDF: Polyvinylidene difluoride
BSA: Bovine serum albumin
TBST: Tris-buffered saline with Tween
ECL: Enhanced chemiluminescence
ATMs: Adipose tissue macrophages
IFN-γ: Interferon-γ
LPS: Lipopolysaccharide
iNOS: Nitric oxide synthase
ROS: Reactive oxygen species
IL-4: Interleukin 4
IL-13: Interleukin-13
STAT1: Signal transducer and activator of transcription 1
JAK1: Janus kinase 1
GAS: γ-Activated sequence
IL-12: Interleukin-12
TNF-α: Tumor necrosis factor-alpha
